# A Prospective Study Comparing Laparoscopic vs. Conventional Stomach Pull Up in Total Pharyngo-Laryngo-Esophagectomy for Post Cricoid Cancer

**DOI:** 10.3390/clinpract11020025

**Published:** 2021-03-29

**Authors:** Basavegowda Vinod Prakash, Ali Zaid Anwar, Mahadev Abhishek, Shivaji Sharma, Saseendran Shruthi

**Affiliations:** Department of Surgical Oncology, Kidwai Memorial Institute of Oncology, M.H. Marigowda Road, Bangalore 560029, India; bvprakashms@gmail.com (B.V.P.); mahadevabhishek@gmail.com (M.A.); shivajisharma6@gmail.com (S.S.); shruthi0908@gmail.com (S.S.)

**Keywords:** post cricoid cancer, laparoscopy, TPLE, stomach pull through

## Abstract

The aim of this study is to compare laparoscopic and conventional techniques following Total Pharyngo-laryngo-esophagectomy (TPLE) with respect to perioperative morbidity and mortality and postoperative recovery in post cricoid cancer patients. This is a prospective study, which was undertaken in Gujrat Cancer Research Institute (GCRI) in the period of July 2007 to March 2010. Fifteen consecutive patients who underwent laparoscopic TPLE were compared to that of 18 consecutive patients who underwent open TPLE. Laparoscopic and open TPLE procedure were compared with respect to patient characteristics, intra operative and complications present. The average duration was observed to be 3.5 h in the MIS (Minimally Invasive Group) group and was 5.3 h in the open group. The average blood loss was 300 mL in the MIS group and 500 mL in the open group. Average duration of the hospital stay in the MIS group was 13 days and 16 days in the open group. In the MIS group, one patient (6.7%) had a pneumonic complication and two patients (13%) had wound complications. In the open group, six patients (33%) had pneumonic consolidation and four patients (22%) had wound infections. In both groups, one patient each suffered mortality. Laparoscopic TPLE has been found to be much safer with less morbidity as compared with open surgery.

## 1. Introduction

Post cricoid cancer is more common in women worldwide and usually associated with iron deficiency anemia and the Plummer-Vinson syndrome. India as well as France have the highest rates of post cricoid cancers throughout the world (annual incidence of 8–15 per 10,000) [1]. Hypopharyngeal cancers usually occur in low socioeconomic classes. Most of the patients usually present late with advanced disease (T3–T4). Generally, prognosis of advanced hypopharyngeal cancer is poor. The prognosis is generally dismal with a mean five-year survival rate of 18–35% (Pingree & Axon) [2,3]. The standard of care is surgery followed by adjuvant radiotherapy. This treatment helps us achieve relief of dysphagia, which is the main symptom, and requires attaining the best possible survival. The quality of life is important in these patients because prognosis is poor. Thus, if the disease can be extirpated with low morbidity, mortality, and a shorter hospital stay, that should be the preferred method of treatment.

Patients requiring circumferential ablative surgery for hypopharynx and cervical esophagus has a poor prognosis and significant morbidity. Hence, the method of reconstruction should be chosen so as to give rise to minimal morbidity, a minimal hospital stay, and allow early return of satisfactory swallowing function. The main goal of treatment is relief of dysphagia and loco-regional control of disease. 

We prefer reconstruction by the stomach pull up because it is easy, reliable, and has acceptable levels of morbidity and mortality. The conventional technique of resection and reconstruction carries significant morbidity because of neck dissection, laparotomy, and trans-hiatal dissection of esophagus, which includes pleural or lung injury, as it is a blind dissection and, thus, causes respiratory morbidity. 

In this context, we contemplated the application of principles of minimally invasive surgery for these particular patient populations. The minimally invasive approach is used to mobilize the stomach and to perform the trans-hiatal dissection of the esophagus, thus, avoiding laparotomy and reducing the morbidity of pain, respiratory complications, and resulting in a better outcome with good quality of life. The minimally invasive surgery is employed without violating the principles of oncologic surgery.

## 2. Materials and Methods

This prospective study compared all patients who underwent laparoscopic Total Pharyngo-laryngo-esophagectomy (TPLE) with patients who underwent open TPLE in the Gujrat Cancer Research Institute (GCRI) operated from July 2007 to March 2010. Fifteen consecutive patients underwent laparoscopic TPLE (Group A) were compared with 18 patients who underwent open TPLE (Group B) from July 2007 to March 2010, i.e., same duration in a single institution. Patients were explained the nature of disease, type of extensive surgery they were subjected to, and the necessity of permanent tracheostoma and loss of voice. Preoperatively, we built the nutritional status through nasogastric tube feeding. All patients for surgery were given antibiotic prophylaxis.

**Figure 1:**
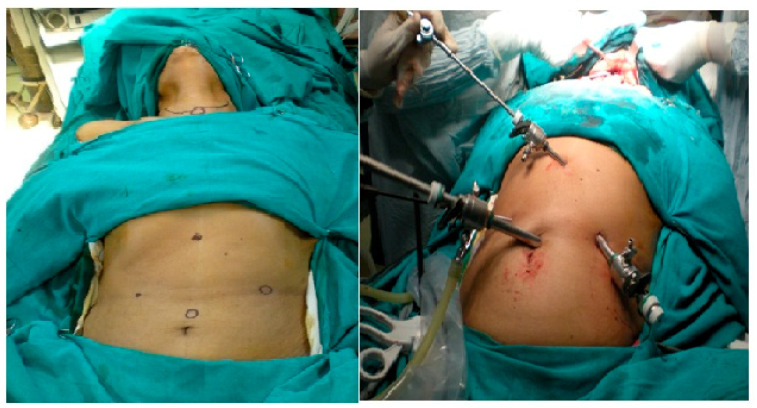
Port placement.

The neck is hyper extended while the patient is in a supine position (Figure 1), subplatysmal flaps were raised, the involvement of internal jugular vein, carotid artery, pre vertebral fascia was excluded, and the modified neck dissection was carried out in N0 disease. In case of palpable nodes, a complete neck dissection was done, the post cricoid tumor was excised inferiorly to the base of tongue, and the distal level of transection is determined by the extent of the disease. If it is limited to above the thoracic inlet, then resection of the larynx, pharynx, and cervical esophagus would be sufficient. If extent of the tumor is below the thoracic inlet, then the entire esophagus is removed. 

In an open method, an upper midline supra-umbilical incision was taken. The greater omentum was mobilized as the left gastro epiploic vessels was ligated and even left gastric vessels were ligated and divided. The preservation of the right gastroepiploic vessels were assured and blind dissection was carried out in the mediastinum through the esophageal hiatus.

In the laparoscopic technique, the peritoneal cavity was insufflated with CO_2_, two 10-mm ports, two 5-mm ports were utilized, and the gastric dissection was completed using a 30-degree telescope in which right gastroepiploic vessels were preserved. The mediastinal dissection was carried out using a 0-degree telescope and carried meticulously to avoid unnecessary injury.

The size of pharyngostoma was estimated and adequate fundal gastrostomy was made and single layer anastomosis was made using interrupted polyglactin suture 2-0. 

**Figure 2:**
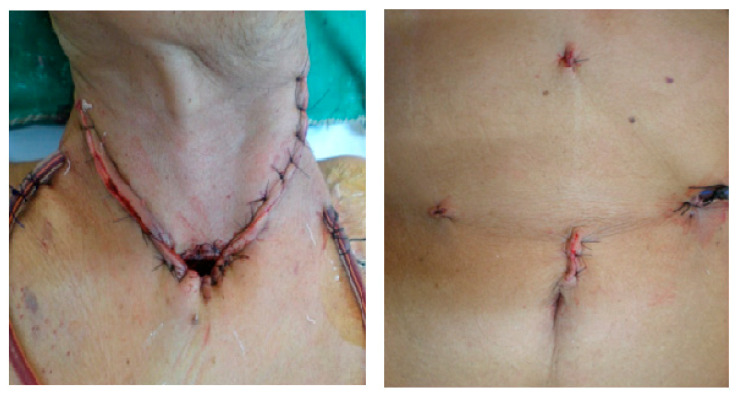
Postoperative.

**Figure 3:**
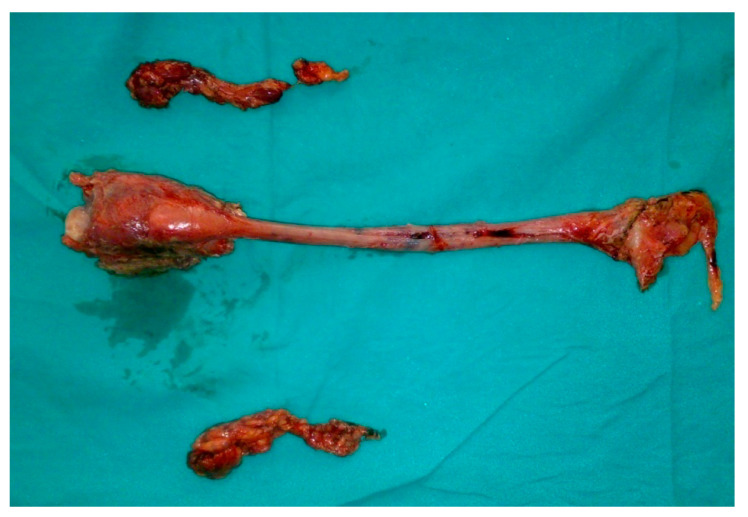
Laryngopharyngoesophagectomy and neck dissection specimen.

A nasogastric tube was placed and feeding jejunostomy was made in all cases, through which enteral nutrition was carried out on the second postoperative day. Each patient was given trial of clear liquids to swallow and observed for anastomotic leakage until the stomach became deflated by an intraoperatively placed nasogastric tube. If a leak or dehiscence occurred, it was managed conservatively. The jejunostomy tube was removed when a patient was able to swallow and retain a full diet. Once histopathology report was available, and standard adjuvant treatment was given according to a unit protocol after postoperative recovery (Figure 2). With respect to pathology, patients with invasive cancer were analyzed to assess the grade of the tumor, nodal status, and margins of resection (Figure 3). Patients were staged using the AJCC (American Joint Committee on Cancer) staging system.

After discharged patients were followed up, during which they were asked for any complaints, they were examined loco-regionally and systemically. They were subjected for routine blood investigation (including TFT, serum calcium), and X-ray chest. The mortality rate related to surgery (within 30 days of surgery) and long-term survival were studied.

All cases were advised for follow-up every two months for the first year, once every three months during the second year, once every four months during the third year, once every six months for the next two years, and then annually. Data collected included patient characteristics, tumor site, and morphology. Operative information included blood loss and duration of surgery. The complications were studied including both major and minor factors.

## 3. Results

In the minimal invasive surgery group, a total of 15 patients underwent surgery, out of which two were male and 13 were female. In the open group, a total 18 patients underwent surgery, out of which 5 were male and 13 were female. The median age was 40 (range 28 to 65) years in laparoscopic TPLE group where in the open group, the median age was 40 (range 21 to 60) years. 

In the MIS (Minimally Invasive Group) group, the most common site was limited to the post-cricoid region (27%) and, in the open group, the most common site of lesion was the hypopharynx and supraglottis in 28% of patients. The average duration of surgery was observed to be 3.86 (range of 3 to 6) hours in the MIS Group. Initially, it was longer in earlier surgeries but, later, it reduces to an average of 3.5 h. The average duration of surgery was observed to be 5.33 (range 4 to 6.5) hours in the Open Group. The difference in the post-operative variables was average blood loss. In the MIS group, it was was 200–500 mL and, in the Open group, it was 300–600 mL. 

The average time taken until postoperative oral intake was 10 (range 6 to 18) days in the MIS group and 10.92 (range 6–19) days in the open group. It took around an average of seven days (4 to 15 days) to remove all drains in both groups. The average duration of the ICU (Intensive Care Unit) stay in the MIS group was 3.4 (1–12) days, and the average duration of the ICU stay in the open group was 4 (2–13) days. The average duration of hospitalization was 13.1 (7 to 37) days in the MIS group and the average duration of hospitalization in the open group was 16.88 (11–32) days.

In the MIS group, one patient (6.7%) had pneumonic consolidation and two patients (13%) had a wound infection. In the open group, six patients (33%) had pneumonic consolidation and four patients (22%) had a wound infection. 

In the MIS group, one perioperative death occurred. This patient had a thoracic duct injury with chyle leak and she expired during re-exploration due to cardiac shock. Whereas in the open group, one patient had wound infection and IJV bleed leading to hemorrhagic shock on the 11th postoperative day.

Regarding the adjuvant therapy, in the MIS group, 10 patients (67%) received post-operative radiotherapy. In the open group, eight patients (44%) received postoperative radiotherapy and five patients (28%) received preoperative radiotherapy while one patient (4%) had received preoperative chemoradiation.

In the MIS group, during the study period, two patients developed cervical LN recurrence and one patient developed lung metastasis. In the open group, one patient developed malignant pleural effusion (Table 1).

## 4. Discussion

Post cricoid cancer is an aggressive cancer. In 1960, Ong and Lee [4] described the use of the transposed stomach to restore gastrointestinal continuity after circumferential pharyngectomy. This method was re-popularized by Orringer [5] after trans-hiatal esophagectomy, which was followed by anastomosis in the neck. Today, a gastric pull-up operation is a preferred technique, especially when a significant tumor extends into the proximal esophagus. The advantage includes dealing with potential skip lesions by total esophagectomy, which is performed as part of the gastric pull-up. The main advantages of the procedure are single stage surgery, and a single well vascularized anastomosis with the stomach. Additionally, stomach pull-up is thought to have less complications than free jejunal transfer (33% vs. 47%, *p* < 0.05). Spiro et al., in 1991 [6], reviewed 120 patients who had gastric transposition from 1973 to 1990 at Memorial Sloan Kettering Cancer Center. There was an 11% operative mortality. Fifty-five percent had intraoperative or perioperative complications. In addition, 13% had anastomotic leaks, and there were three instances of stomach necrosis. This translates to a median of eight days of a hospital stay when recovery was uneventful and 11 days when there were associated complications. They concluded that gastric pull-up is a reliable, reconstructive method, but advocates careful patient selection to minimize morbidity. According to another study, there was no significant difference with regard to the survival between gastric transposition and free jejunal autograft, but there were fewer respiratory complications (33% vs. 47%, *p* < 0.05), significantly low local recurrences (15.8% vs. 33.8%, *p* = 0.004), and higher survival without dysphagia (76% vs. 89%, *p* < 0.05) [6,7].

According to Schusterman et al. [8], in patients with advanced cancer, extensive esophageal resection into the chest is often required, and gastric pull-up seems to be an easier and more direct form of reconstruction. Wong et al. [9] have, until now, reported the largest experience on a minimal invasive approach of gastric mobilization and esophageal dissection on 13 TPLE of which nine were done totally laparoscopically and four were performed hand assisted. This series confirmed the feasibility of the laparoscopic approach and demonstrated its safety. The mean operative time was 8.5 h (range: 5–11), there was no 30-days mortality, and the morbidity rate was 42%, which was more favorable in comparison with their open approach. The mean hospital stay was 41 (range: 18–75) days in this Wong et al. study.

One series by Rossi et al. [10] have also reported this technique on four patients with recurrent disease after primary chemo-radiotherapy. The mean operative time was 345 min (range: 300–384). The hospital stay was 20 days (18–20).

In our series, the mean duration of surgery was 4 h, of which the mean time taken for laparoscopy was 3 h (180 min). However, as the experience increased in the last two cases, it was 2 h (120 min). This observation is comparable to what was reported by Wong et al. [9] in which the median total operative time was 8.5 h (range, 5–11 h) and the laparoscopic time was less than 4 hours. The mean duration of hospital stay in our patients was 16 days (7–37 days) which is comparable to what was reported by Rossi et al. [10] and less than Wong et al. [9] where the mean hospital stay was 41 (range: 18–75) days. 

We have noticed that the operative time was less in the patients who were operated later on in the series because of a learning curve associated with this procedure in the earlier cases.

The laparoscopic TPLE group lost less blood and, hence, had fewer requirements for transfusion during the admission than the open approach group. The estimated average blood loss in the laparoscopic TPLE was 300 mL (200–500), which is less than the conventional open procedure (i.e., 460 mL (300–600)) in our series. Similar observations were made by Wong et al. [9], which had less operative blood loss. In a report summarizing nationwide statistics, the mortality rates from TPLE ranged from 3.4% in high-volume centers to as high as 17.3% in low-volume centers. Others have also reported high rates of morbidity (60%–84%) and mortality (1%–4%) [11,12]. Much of the morbidity of the procedure including lung complications, cardiopulmonary failure, complications from delayed mobilization, and wound complications are due to the method of access. Most patients experience less pain, fewer wound complications, less blood loss, and a quicker return to normal activity with a laparoscopic group.

In our laparoscopic TPLE (Total Pharyngo Laryngo Oesophagectomy) group, 73% of patients had postoperative morbidity. Among them, one patient (6.7%) developed pneumonic consolidation and wound infection was seen in two patients (13%). Similarly to an open group, 89% of the patients developed morbidities, which included wound infection in four (22%) patients, leak in one patient (5.5%), and consolidation in six patients (33%). This suggests that a significant reduction in respiratory complications with a laparoscopic TPLE group (6.7%) occurred as compared to an open group (33%). The wound complication rate was also reduced in the laparoscopic TPLE group. Other studies have also shown higher incidence of pneumonia in an open group as compared to laparoscopic TPLE. 

The quality of life of patients undergoing laparoscopic TPLE improved, as shown by Wong et al. [9]. Additional theoretical advantages of the use of laparoscopy in cancer treatments result from a decreased surgical stress response, which may minimize immunologic suppression. Once again, this is a possible consideration for long-term survival. 

By comparing laparoscopic TPLE with an open approach in our experience, we observed that laparoscopic TPLE for post-cricoid cancer was a safe option in experienced hands. Since this procedure is associated with a learning curve, our results will improve with time, as has been observed by others. Laparoscopic TPLE has the potential to replace conventional techniques.

## 5. Conclusions

A minimally invasive technique for pulling up the stomach in patients suffering from post-cricoid cancer and is considerably safe as compared to an open technique. With regard to morbidity and mortality showing favorable results with the laparoscopic group since it has less operative blood loss, decreased respiration complication, and decreased wound infection.

Hence, the laparoscopic technique is superior when compared to an open technique and has clear advantages in spite of the difficulty in the learning curve present and can be collectively encouraged.

## Figures and Tables

**Table 1:** Post-operative complication.

Complication	MIS Group (15)	Open Group (18)
Pulmonary	1 (6.7%)	6 (33%)
Wound infection	2 (13%)	4 (22%)
Hypothyroidism	4 (27%)	6 (33%)
Hypoparathyroidism	7 (47%)	4 (22%)
Anastomotic Leak	1 (6.7%)	1 (5.5%)
Anastomotic stricture	0 (0%)	1 (5.5%)
IJV bleed	1 (6.7%)	2 (11%)
Chyle leak	1 (6.7%)	1 (5.5%)
